# Flooding and herbivory: the effect of concurrent stress factors on plant volatile emissions and gene expression in two heirloom tomato varieties

**DOI:** 10.1186/s12870-022-03911-3

**Published:** 2022-11-17

**Authors:** Esther Ngumbi, Erinn Dady, Bernarda Calla

**Affiliations:** 1 Department of Entomology, University of Illinois at Urbana-Champaign, Urbana, IL 61801 USA; 2 USDA-ARS Forage Seed and Cereal Research Unit, Corvallis, OR 97331 USA

**Keywords:** Flooding stress, Herbivory, Gene expression, Heirloom tomatoes, Combined stresses, Phytochemistry, Beet Armyworm, Volatile Organic Compounds

## Abstract

**Background:**

In nature and in cultivated fields, plants encounter multiple stress factors. Nonetheless, our understanding of how plants actively respond to combinatorial stress remains limited. Among the least studied stress combination is that of flooding and herbivory, despite the growing importance of these stressors in the context of climate change. We investigated plant chemistry and gene expression changes in two heirloom tomato varieties: Cherokee Purple (CP) and Striped German (SG) in response to flooding, herbivory by *Spodoptera exigua*, and their combination.

**Results:**

Volatile organic compounds (VOCs) identified in tomato plants subjected to flooding and/or herbivory included several mono- and sesquiterpenes. Flooding was the main factor altering VOCs emission rates, and impacting plant biomass accumulation, while different varieties had quantitative differences in their VOC emissions. At the gene expression levels, there were 335 differentially expressed genes between the two tomato plant varieties, these included genes encoding for phenylalanine ammonia-lyase (PAL), cinnamoyl-CoA-reductase-like, and phytoene synthase (Psy1). Flooding and variety effects together influenced abscisic acid (ABA) signaling genes with the SG variety showing higher levels of ABA production and ABA-dependent signaling upon flooding. Flooding downregulated genes associated with cytokinin catabolism and general defense response and upregulated genes associated with ethylene biosynthesis, anthocyanin biosynthesis, and gibberellin biosynthesis. Combining flooding and herbivory induced the upregulation of genes including chalcone synthase (CHS), PAL, and genes encoding BAHD acyltransferase and UDP-glucose iridoid glucosyltransferase-like genes in one of the tomato varieties (CP) and a disproportionate number of heat-shock proteins in SG. Only the SG variety had measurable changes in gene expression due to herbivory alone, upregulating zeatin, and O-glucosyltransferase and thioredoxin among others.

**Conclusion:**

Our results suggest that both heirloom tomato plant varieties differ in their production of secondary metabolites including phenylpropanoids and terpenoids and their regulation and activation of ABA signaling upon stress associated with flooding. Herbivory and flooding together had interacting effects that were evident at the level of plant chemistry (VOCs production), gene expression and biomass markers. Results from our study highlight the complex nature of plant responses to combinatorial stresses and point at specific genes and pathways that are affected by flooding and herbivory combined.

**Supplementary Information:**

The online version contains supplementary material available at 10.1186/s12870-022-03911-3.

## Background

Within natural environments and in cultivated fields, plants often encounter diverse biotic and abiotic stressors such as herbivory, pathogen attack, drought, and flooding occurring individually, sequentially, or simultaneously [1–12]. To survive and reproduce, plants must appropriately respond to these stress factors [13]. Available evidence shows that the response of plants to stress combinations is unique and that it cannot be extrapolated from responses to individual stresses [4, 9, 11, 14–17]. When two or more stresses occur simultaneously, their effects can be additive, synergistic, or neutral. In some cases, the influence of one stress can become dominant [17]. The overall effect of stress combinations is further dependent on plant species, genotype, specific herbivore species, and severity of the co-occurring stresses [4, 11]. Numerous studies of combinatorial stresses have focused on interactions between abiotic and biotic stressors; for example: drought and herbivory [9, 18–20], drought and pathogen infection [10], salinity and ozone (O_3_) exposure [21], salinity and pathogen infection [21], ozone exposure and herbivory [22], elevated CO_2_ and herbivory [23], and high temperature and herbivory [9]. Despite the growing importance of flooding and herbivory in the context of climate change, the combined effects of these stressors are understudied.

Across North America, and in many regions of the world, flooding and waterlogging have become more frequent, severe, and economically damaging, with impacts on crops comparable in magnitude to drought and extreme temperatures [24, 25]. Waterlogging occurs when water is unable to drain away, leading to soil that is fully saturated. Flooding occurs when soil and roots are completely submerged under water. Both conditions challenge plants, due to oxygen deprivation in plant roots, which directly affects normal plant biochemical, molecular, and physiological processes [26–29]. Flooding may occur simultaneously or sequentially with biotic stressors such as insect herbivory [5, 21, 30, 31]. The simultaneous occurrence of flooding stress with herbivory may modify plant stress responses.

Genome-wide transcriptomic profiling studies on plants under combinations of biotic and abiotic stresses have been conducted in the past. For example, Rasmussen et al., [15] analyzed differences in gene expression patterns in ten *Arabidopsis thaliana* ecotypes challenged by single or dual (a)biotic stress combinations. Results from that study showed that changes in gene expression in response to combined stresses could not be predicted from single-stress treatments. Nguyen et al., [19] reported that flooding or drought pretreatment significantly modified the transcriptome signature of *Solanum dulcamara* plants that were already infested with *S. exigua*. Suzuki et al., [9] reviewed 33 different studies involving combinatorial stresses and found that simultaneous occurrence of different stresses generated unique responses, suggesting that plants dynamically respond to multiple co-occurring stress factors. Tamang et al., [32] reported that in soybeans, drought and flooding resulted in stress-specific and overlapping transcriptomic responses. Taken together, these studies suggest that transcriptomic responses/gene expression patterns of plants to combinatorial stresses cannot be predicted from responses to individual stresses.

Tomato (*Solanum lycopersicum* L.) is one of the most important vegetable crops in the world because of its high lycopene content, anti-oxidative properties, and as a source of micronutrients [33]. In 2018, globally, over 186 million tons of tomato were produced, worth over US $60 billion [34]. In addition, since the completion of its genome, tomato has been widely used as a model plant for studies investigating crop plant responses to biotic and abiotic stress [19, 33, 35–38] including flooding [29, 33, 39, 40]. Safavi-Rizi et al., [33], for example, investigated the effects of flooding stress associated hypoxia on the regulation of gene expression in tomato roots. They demonstrated transcriptome reprogramming in response to flooding stress and identified novel genes and pathways that potentially contribute to flooding stress tolerance. De Ollas et al., [29] investigated the role of abscisic acid (ABA) on the regulation of genetic and metabolic responses of tomato to soil flooding. They showed that ABA depletion in waterlogged tomato tissues acts as a positive signal, inducing several specific genetic and metabolic responses to flooding. In another study, Rodriguez-Saona et al., [41] investigated transcriptome changes in tomato in response to feeding by the potato aphid, (*Macrosiphum euphorbiae*), and the beet armyworm (*Spodoptera exigua*), individually or in combination. They demonstrated that herbivory resulted in the upregulation of several defense-related genes including genes encoding for threonine deaminase and numerous protease inhibitors. Considering the global diversity in the tomato germplasm and the contrasting and inherently complex and unpredictable combinations of stresses, there is an urgent need for studies aimed to understand how different tomato varieties diverge in their chemical and molecular response to stress combinations.

In this study, we investigated the effects of flooding, herbivory and their interaction on plant growth and plant chemistry and performed an unsupervised exploratory analysis of gene expression patterns. Our study focuses on two heirloom tomato varieties responding to waterlogging and/or herbivory by *Spodotera exigua* (the beet armyworm) larvae. We hypothesized that: 1) the combined stress of flooding and herbivory alters plant chemistry and negatively impacts plant growth, with a different effect than either of flooding or herbivory alone; and 2) the altered plant chemistry elicited during combinations of flooding and herbivory has a molecular underpinning detectable as differential gene expression.

## Results

### Volatile organic compounds

**Fig. 1:**
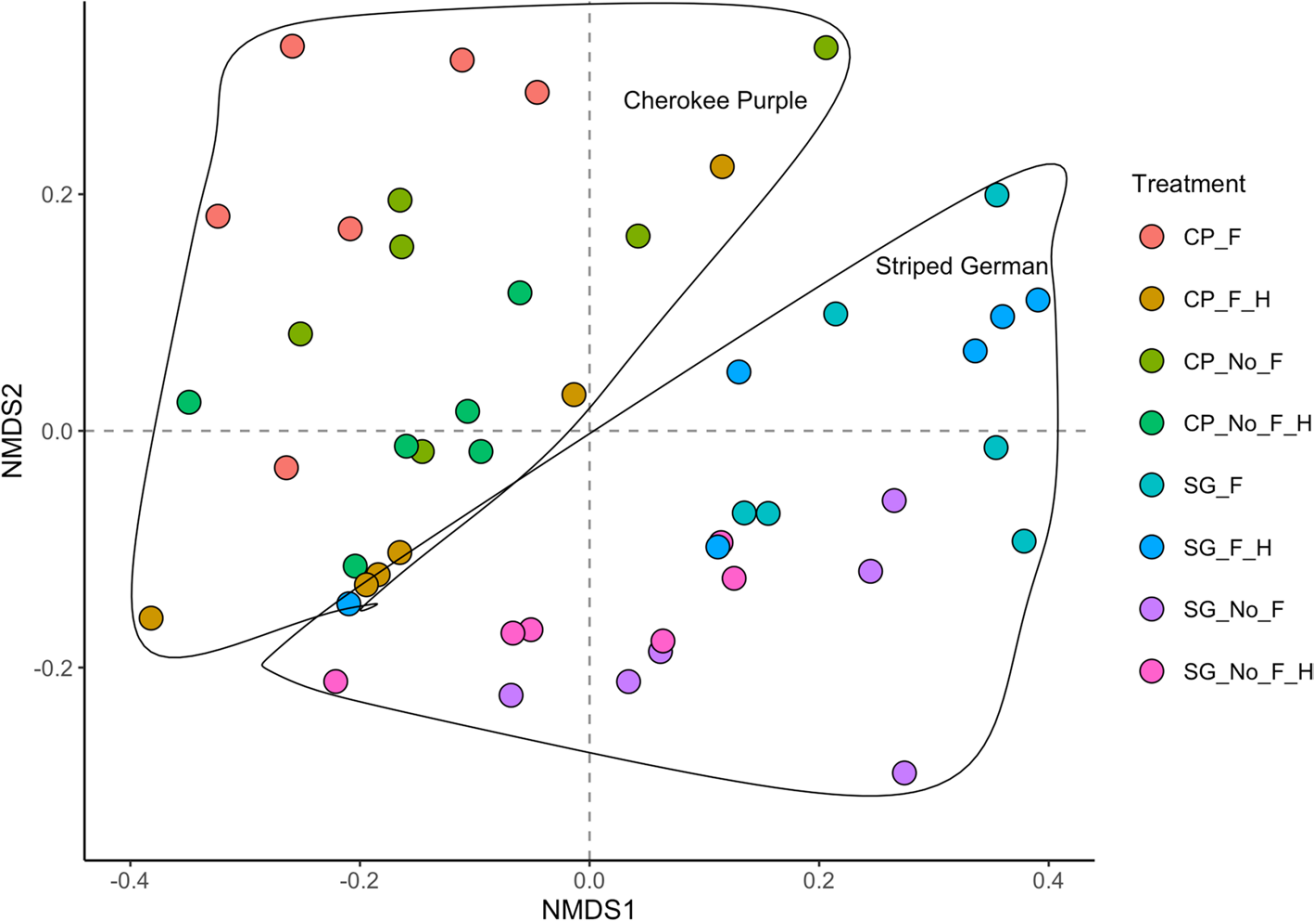
Non-metric multidimensional scaling (NMDS) ordination constructed with a Bray–Curtis dissimilarity matrix of total volatile organic compounds emitted by two tomato varieties, Cherokee Purple (CP) and Striped German (SG), exposed to the following stress factors: (1) no flooding [No_F], (2) no flooding + herbivory [No_F_H], (3) flooding [F], and (4) flooding + herbivory [F_H]

A total of 18 volatile organic compounds (VOCs) were detected and quantified from the headspace of the two heirloom tomato varieties subjected to four stress factors: (1) no flooding and no herbivory (control), (2) no flooding + herbivory, (3) flooding, and (4) flooding + herbivory. The detected VOCs, the majority of which were terpenoids, included the monoterpenes: α-pinene, o-cymene, σ-cymene, β-pinene, ( +)-4-carene, α-terpinene, β-phellandrene, trans-β-ocimene, p-cymene, β-ocimene, gamma-terpinene, and α-terpinolene; the sesquiterpenes: p-cresol (phenol), δ-elemene, β-elemene, caryophyllene, and humulene; and the alkane hydrocarbons: 1-pentadecane, and pentadecane. The non-metric dimensional scaling (NMDS) analysis of VOC emissions on Bray–Curtis dissimilarity matrix revealed strong clustering due to variety (Fig. 1).

**Fig. 2:**
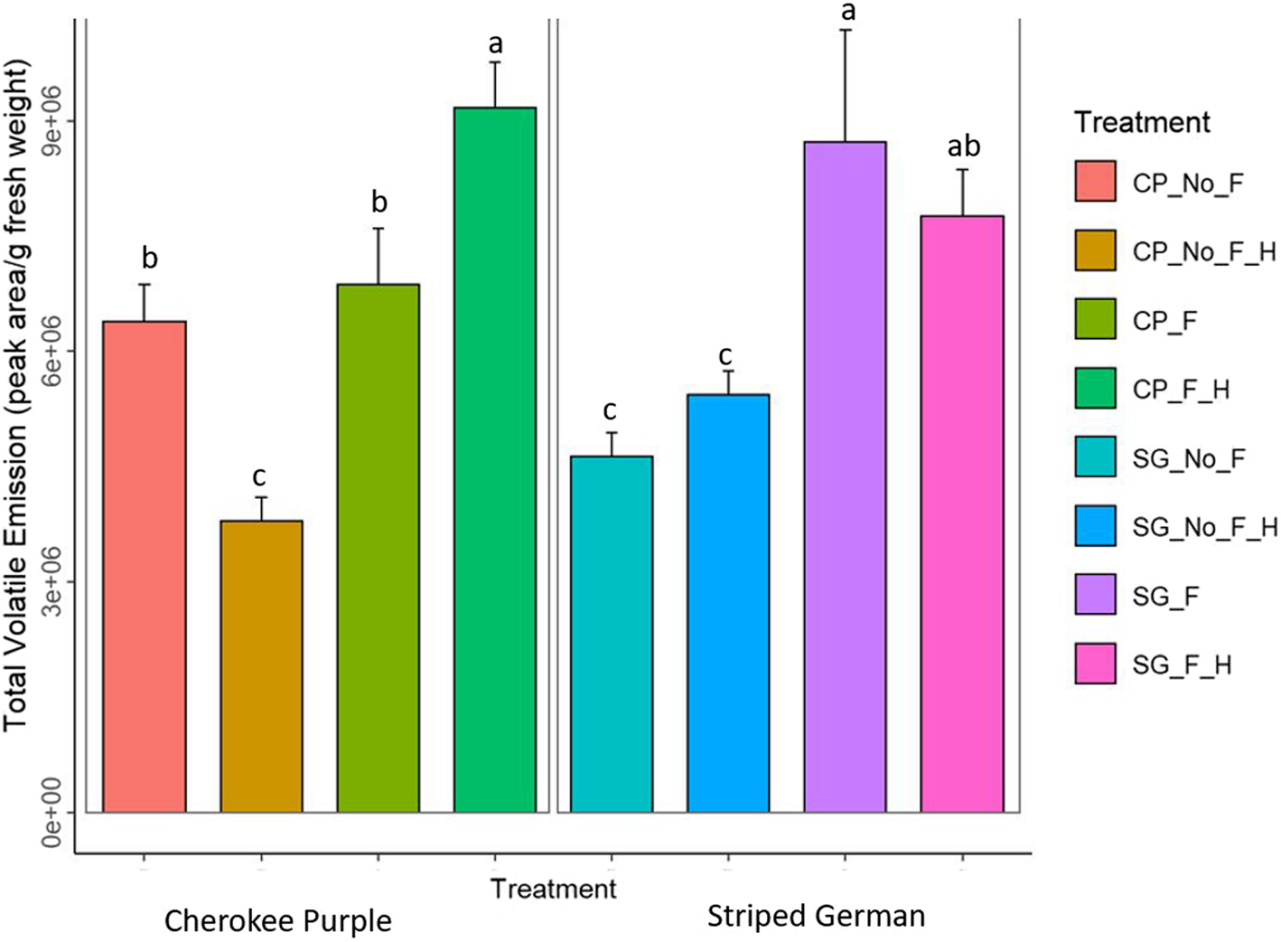
Total volatile organic compounds emissions in two heirloom tomato varieties, Cherokee Purple (CP) and Striped German, (SG) exposed to the following stress factors: (1) no flooding [No_F], (2) no flooding + herbivory [No_F_H], (3) flooding [F], and (4) flooding + herbivory [F_H]. Tukey’s honest significance test was used to group means. Bars represent mean ± SE. Means with different letters are different (as determined by Tukey HSD, *P* < 0.05)

Three-way analysis of variance (ANOVA) revealed that total volatile organic compound emissions in the two heirloom varieties was influenced by flooding F_[flooding]_ = 38.53, *P*_[flooding]_ =  < 0.0001 and the interaction of variety, flooding, and herbivory F_[variety*flooding*herbivory]_ = 11.34, *P*_[variety*flooding*herbivory]_ = 0.0017) (Table 1; Fig. 2). The highest VOCs emission rates, for both varieties, were recorded in the flooding treatments and the treatment involving the combinatorial stress of flooding and herbivory (Fig. 2). When considering the total volatile organic compounds emitted in plants exposed to flooding only, the Striped German (SG) variety emitted more volatiles than Cherokee Purple (CP) (Fig. 2).

**Table 1:** Three-way analysis of variance table of treatment effect (variety, flooding, herbivory, and their interactions) on total volatile emissions

Factors	DF	SS	MS	F- Value	*P*-Value
Variety	1	8.329e + 10	8.329e + 10	0.028	0.8671
Flooding	1	1.133e + 14	1.133e + 14	38.536	< .0001***
Herbivory	1	1.698e + 11	1.698e + 11	0.058	0.8112
Variety x Flooding	1	2.535e + 11	2.535e + 11	0.086	0.7705
Variety x Herbivory	1	1.696e + 10	1.696e + 10	0.006	0.9382
Flooding x Herbivory	1	7.350e + 12	7.350e + 12	2.501	0.1216
Variety x Flooding x Herbivory	1	3.332e + 13	3.332e + 13	11.337	0.0016**
Residuals	40	1.176e + 14	2.939e + 12

According to the random forest (RF) analysis, that ranked individual compounds by their importance in contributing to treatment separation and differences, the most important compounds were α-terpinolene, ( +)-4-carene, δ-elemene, α-pinene, humulene, β-phellandrene, caryophyllene, o-cymene, β-pinene and p-cymene (Table 2).

**Table 2:** Ranking of volatile organic compounds (VOCs) that contribute to treatment differences. Random Forest was used. Compound rankings were determined using Mean Decrease Accuracy (MDA)

Rank	Volatile Organic Compound	Mean Decrease Accuracy
1	p-cymene	51.285543
2	β-pinene	48.739645
3	o-cymene	44.327346
4	Caryophyllene	43.056983
5	β-phellandrene	42.558952
6	Humulene	41.359839
7	α-pinene	38.821284
8	δ-elemene	37.039133
9	( +)- 4-carene	35.596207
10	α-terpinolene	33.440834
11	α-terpinene	29.718382
12	gamma-Terpinene	22.976904
13	β-elemene	19.581877
14	Pentadecane	19.326385
15	p-cresol	13.409866
16	1-Pentadecane	12.609018
17	β-ocimene	12.549250
18	trans-β-ocimene	-0.951859

Results of three-way ANOVA of the effects of flooding, herbivory, variety, and their interactions on plant biomass characteristics (root length, root wet weight, root dry weight, shoot diameter, shoot length, shoot dry weight, and shoot dry weight) revealed significant differences for the main effects of variety and flooding on root length, root wet weight, root dry weight, shoot wet weight and shoot dry weight (Table 3). Flooding negatively affected plant growth. Wet root weight and dry root weight were significantly reduced by flooding in both heirloom varieties. Shoot dry weight was significantly reduced by flooding and by the interaction between flooding and herbivory in Striped German.

**Table 3:** Three-way analysis of variance table of treatment effect (variety, flooding, herbivory, and their interactions) on plant growth characteristics

Main Factor	Variety	Flooding	Herbivory	Var*F	Var*H	F*H	Var*F*H
Degrees of Freedom (Num, Den)	1,40	1,40	1,40	1,40	1,40	1,40	1,40
Parameter	F value and *P* value in parenthesis						
Root length (cm)	18.125 (< .0001)	27.626 (< .0001)	8.619 (0.0054)	6.214 (0.0169)	0.210 (0.6494)	0.812 (0.3729)	5.630 (0.0225)
Root wet weight (g)	30.812 (< .0001)	21.703 (< .0001)	2.044 (0.1605)	4.811 (0.0341)	0.370 (0.5466)	11.037 (0.0019)	4.921 (0.0322)
Root dry weight (g)	19.754 (< .0001)	80.200 (< .0001)	0.599 (0.4435)	2.465 (0.1243)	3.368 (0.0739)	1.941 (0.1713)	0.669 (0.4181)
Shoot diameter (cm)	0.849 (0.362)	45.660 (< .0001)	0.377 (0.542)	0.094 (0.760)	0.849 (0.362)	1.509 (0.226)	2.358 (0.132)
Shoot length (cm)	4.751 (0.0352)	78.294 (< .0001)	0.026 (0.8737)	7.233 (0.0103)	4.160 (0.0480)	8.234 (0.0065)	5.427 (0.0249)
Shoot wet weight (g)	11.243 (0.0017)	51.546 (< .0001)	0.255 (0.6166)	6.606 (0.0139)	6.311 (0.0161)	2.396 (0.1295)	3.812 (0.0579)
Shoot dry weight (g)	27.951 (< .0001)	43.579 (< .0001)	0.022 (0.8836)	11.327 (0.0017)	2.163 (0.1492)	2.740 (0.1057)	2.568 (0.1169)

### Gene expression analysis

Sequencing resulted in over 900 million high-quality reads (Phred score > 35 average along entire read), with an average of 37 million reads per library. There were 826 genes changing in expression between either of the factors analyzed and their interactions (fold change > 4, FDR-corrected *p*-value < 0.01).

### Differential expression between varieties and its interaction with flooding

To investigate specific genes that are more likely to be differentially expressed between the two varieties, a more stringent cut off was used (fold-change > 8 *p*-val < 0.01) limiting the analysis to transcripts with very high differential expression and looking at the contrasts between varieties. This resulted in a set comprising 335 genes (Table S1) that were further used in a cluster analysis.

**Fig. 3:**
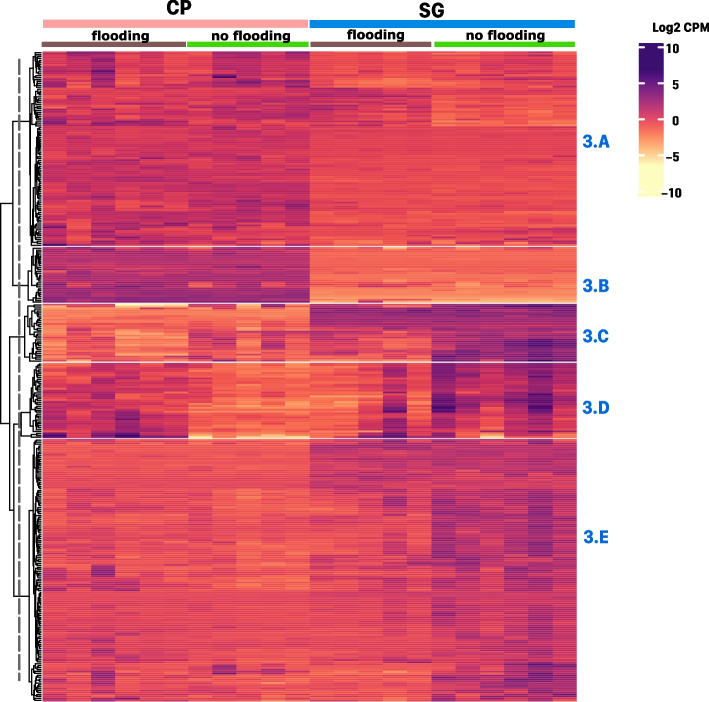
K-means clustering analysis of genes differentially expressed between two heirloom tomato varieties (fold-change > 8 and; FDR *p*-value < 0.01). CP: Cherokee Purple, SG: Striped German. The heatmap has treatments as columns and genes as rows. The top right inset shows the color scale for log2 counts per million (CPM) values with purple tones for up-regulated genes and yellow tones for down-regulated genes. The vertical striped line on dendrogram shows the level at which the tree was cut to separate groups based on k-means. Each separate cluster was named according to figure number followed by and alphabetic character. Each cluster gene content is described in detail in the text

K-means clustering was applied to both: samples and genes. Clustering of samples confirmed two outlier samples that were further removed. The clustering separated five distinct groups of genes, designated 3.A, 3.B, 3.C, 3.D and 3.E (Fig. 3). Clusters 3.A and 3.B contained genes with higher expression in CP than in SG. There were 28 genes in cluster 3.B, a cluster showing marked and consistent high differences between CP and SG. Genes in this cluster included two phenylalanine ammonia-lyase (PAL), the enzyme in charge of the first committed step in the phenylpropanoid pathway. Also in the cluster were a gene encoding cinnamoyl-CoA reductase-like (LOC101262601), and one encoding phytoene synthase (Psy1) which catalyzes the first step in the carotenoid biosynthetic pathway. The top GO biological process terms in clusters A and B combined included: cinnamic acid biosynthetic process (GO:0009800), L-phenylalanine catabolic process (GO:0006559), trans-zeatin biosynthetic process (GO:0033466), glutamine family amino acid catabolic process (GO:0009065), and gibberellin biosynthetic process (GO:0009686) (Table S2). Taken together, the data suggests that both varieties might constitutively differ in the production of secondary metabolites including phenylpropanoids and terpenoids, where CP might have higher constitutive production of these compounds.

Clusters 3.C and 3.E contained genes with lower expression in CP compared to SG (Fig. 3). Cluster 3.C had the most marked differences and comprised 29 genes. Genes in cluster 3.C with the most consistent and larger differences between both varieties (first half in top of the cluster) include several uncharacterized genes. GO biological processes terms in clusters 3.C and 3.E were enriched for disparate terms ranging from photosynthesis, secondary metabolism, and metabolism of high-molecular weight sugars (Table S1, Table S2).

Cluster 3.D contains transcripts that were affected by the interaction of flooding and variety where the flooding treatment most extremely affected varieties in opposite ways, with transcripts increasing in expression upon flooding in CP but decreasing in SG. Cluster 3.D contained 38 genes including 10 uncharacterized products and several transcription factors. The cluster is enriched for biological processed related to abscisic acid (ABA). Genes include several negative regulators of ABA signaling and genes known to respond to increased ABA in the cell environment. CP leaves appear to be responding to root flooding by repressing ABA production. SG, in turn, might have constitutively high ABA production or ABA-dependent signaling (Table S1, Table S2).

#### Changes in gene expression due to flooding

**Fig. 4:**
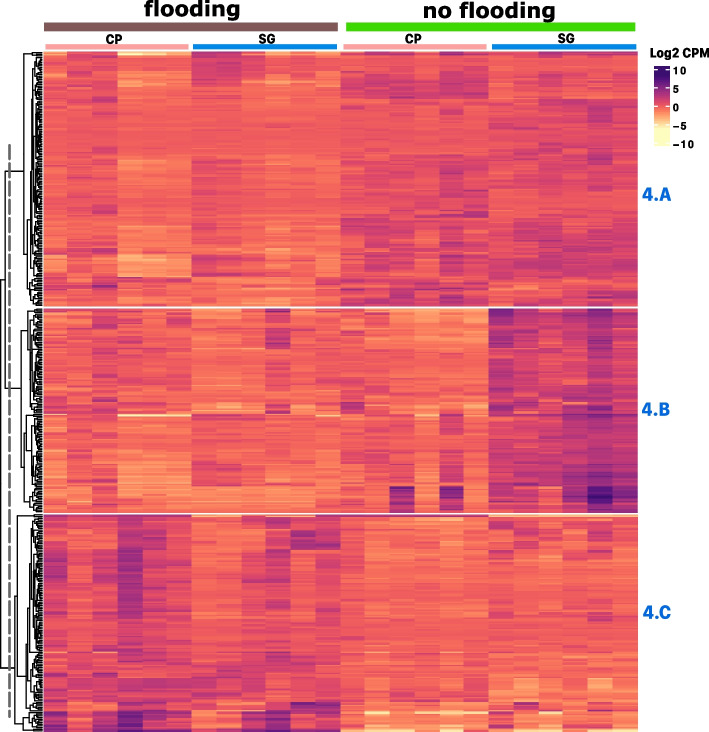
K-means clustering analysis of genes differentially expressed between flooded tomato plants and non-flooded controls (fold-change > 8 and; FDR *p*-value < 0.01). CP: Cherokee Purple, SG: Striped German. The heatmap has treatments as columns and genes as rows. The top right inset shows the color scale for log2 counts per million (CPM) values with purple tones for up-regulated genes and yellow tones for down-regulated genes. The vertical striped line on dendrogram shows the level at which the tree was cut to separate groups based on k-means. Each separate cluster was named according to figure number followed by and alphabetic character. Each cluster gene content is described in detail in the text

A subset of 404 genes were identified as highly differentially expressed at fold-change > 8, FDR *p*-value < 0.01 (Table S1) using contrasts between flooded and non-flooded samples. Clustering of samples successfully separated both treatments, and clustering of genes resulted on three distinct gene clusters (Fig. 4). Roughly, the subcluster (4.A) contains 152 genes that were generally downregulated with flooding, this cluster is significantly enriched for GO biological terms in cytokinin catabolism and general defense response (Table S2, Table S3). The differences in response to flooding between both varieties is evident in Cluster 4.B, with 122 genes, it includes genes that were downregulated in SG upon flooding. The top GO biological process term that was significantly enriched in 4.B was photosynthesis and the second was ABA signaling. Other GO biological processes that were enriched comprised response to water deprivation (GO:0009414), and wounding (GO:009611). There were two zeatin O-glucosyltransferase and one zeatin O-xylosyltransferase (Table S2). In general, this subcluster shows that varieties had different response to flooding stress.

Finally, cluster 4.C contained genes that were upregulated with flooding in both varieties and with no evident effect of herbivory (Fig. 4). Thus, this sub-cluster represents the general response to flooding. In this set of 130 genes, there were two genes encoding for enzymes in the first dedicated steps in the biosynthesis of ethylene: 1-aminocyclopropane-1-carboxylate synthase 3 (ACS3) and 1-aminocyclopropane-1-carboxylate oxidase homolog (ACO3), and. Increased ethylene biosynthesis is a well-documented process in waterlogged plants, including tomato [27, 39, 42]. We could also identify four genes in the flavonoid pathway that indicate increased anthocyanin production: a putative flavonoid 3′5' hydroxylase (LOC100736504), dihydroflavonol 4-reductase (LOC544150), a flavonoid 3',5'-methyltransferase (AnthOMT), and leucoanthocyanidin dioxygenase (LOC101251607). Notably, the set also comprised four genes encoding enzymes in the latest oxygenation steps of gibberellic acid (GA) that give rise to active gibberellin forms: gibberellin 2-beta-dioxygenase 1 (LOC101249786), gibberellin 2-oxidase 2 (GA2ox2), gibberellin 20-oxidase-3 (gene-20ox-3), and gibberellin 3-beta-dioxygenase 1-like (LOC101257892). The most significantly enriched GO biological process term was “protein folding” (GO:0006457), followed by “fruit ripening” (GO:0009835), and anthocyanin-containing compounds (GO:0009718) (Table S3). Protein folding enrichment is the result of the presence of eight (8) heat-shock proteins in the set. Heat-shock proteins are also markers of defense and response to various stresses.

#### Changes in gene expression due to herbivory

The response to herbivory was the weakest. Using the stringer criteria as in previous sections, only 36 genes were differentially expressed between plants responding to *S. exigua* feeding and no herbivory controls. Clustering analysis of this set did not show clear partitioning between treated and control samples. To further characterize the effects of herbivory, the analysis was repeated with the less stringent cut-off (fold-change > 4 FDR-corrected *p*-value < 0.01) and separately within each variety.

### Gene expression due to herbivory in the Cherokee Purple variety

**Fig. 5:**
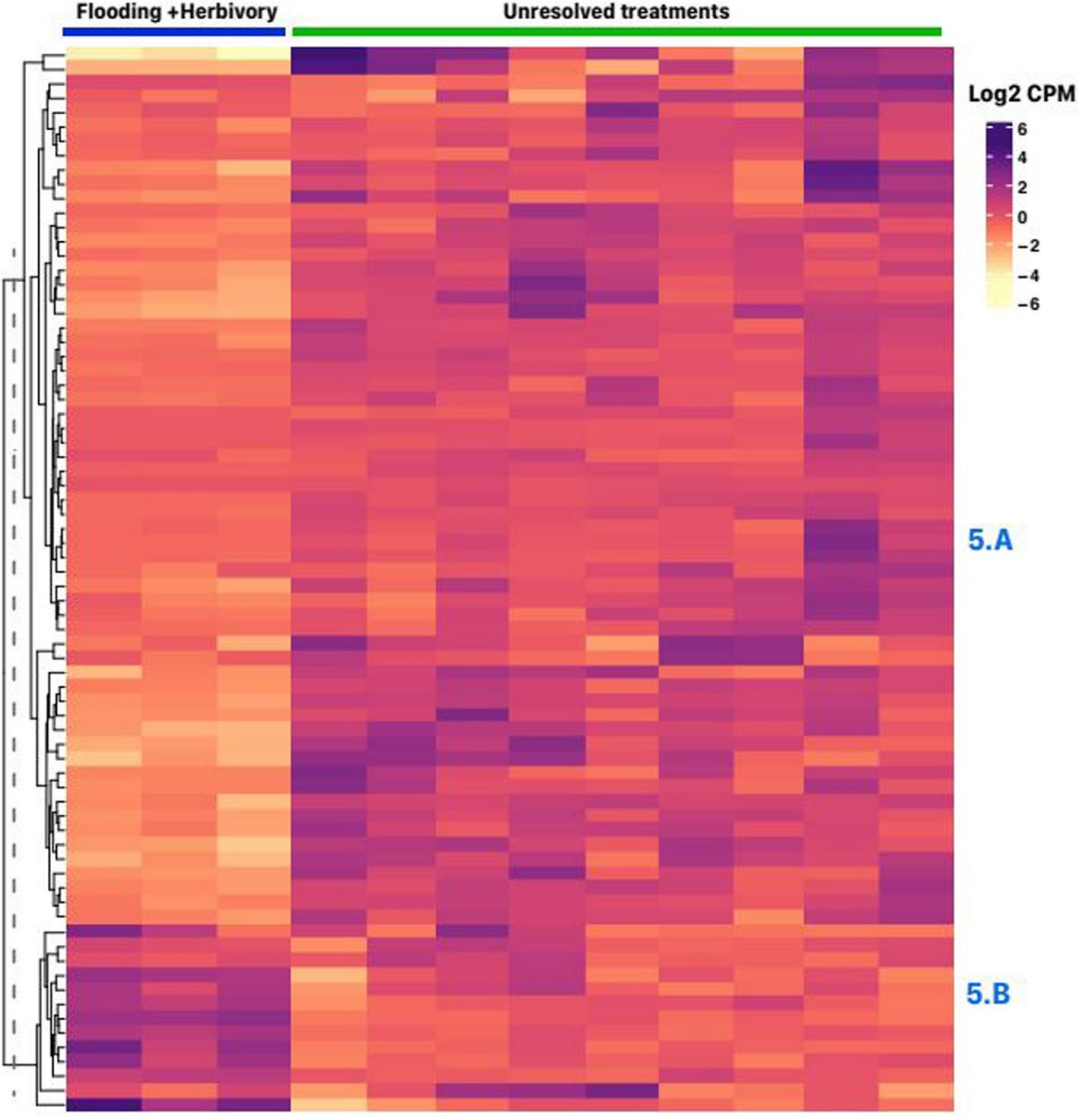
K-means clustering analysis of genes differentially expressed in leaf tissue of tomato plants challenged with herbivory and controls in the tomato variety “Cherokee Purple” (fold-change > 4 and; FDR *p*-value < 0.01). The heatmap has treatments as columns and genes as rows. The top right inset shows the color scale for log2 counts per million (CPM) values with purple tones for up-regulated genes and yellow tones for down-regulated genes. The vertical striped line on dendrogram shows the level at which the tree was cut to separate groups based on k-means. Each separate cluster was named according to figure number followed by and alphabetic character. Each cluster gene content is described in detail in the text

Seventy-four genes were differentially expressed between samples subjected to herbivory and controls in CP. The effect of herbivory was evident in flooded samples only (Fig. 5), and only two gene clusters could be distinguished in this set: Cluster 5.A, with 61 genes consistently downregulated upon herbivory, and Cluster 5.B with 13 genes that were upregulated with herbivory (different only in flooded samples). The top significantly enriched GO biological process in cluster 5.A was lignin biosynthesis (GO:0009809) owing the presence of transcripts encoding caffeoylshikimate esterase (LOC101259602), cinnamyl alcohol dehydrogenase (LOC101245999), caffeoyl-CoA O-methyltransferase (LOC101265977), among others. Cluster 5.A also include five transcripts encoding proteins from the plant GDSL esterase/lipase superfamily (CD1, LOC101262291, LOC101251962, LOC101266652, and LOC101267033) (Tables S1, S2 and S3); these hydrolytic enzymes have been implicated in the response to several biotic and abiotic stress response in plants [43].

Cluster 5.B includes chalcone synthase (CHS1) and PAL (LOC112941051) indicating up-regulation of the phenylpropanoid pathway. A gene encoding for a BAHD acyltransferase (gene-LOC101256185) was also present in the set. BAHD acyltransferase acylates phenolics compounds to change their transporting and reactivity properties. Also in the set was a UDP-glucose iridoid glucosyltransferase-like, suggesting the involvement of iridoid terpenes in the response to herbivore in flooded samples. In addition, there were two heat-shock proteins in cluster 5.B.

### Gene expression due to herbivory in the Striped German variety

**Fig. 6:**
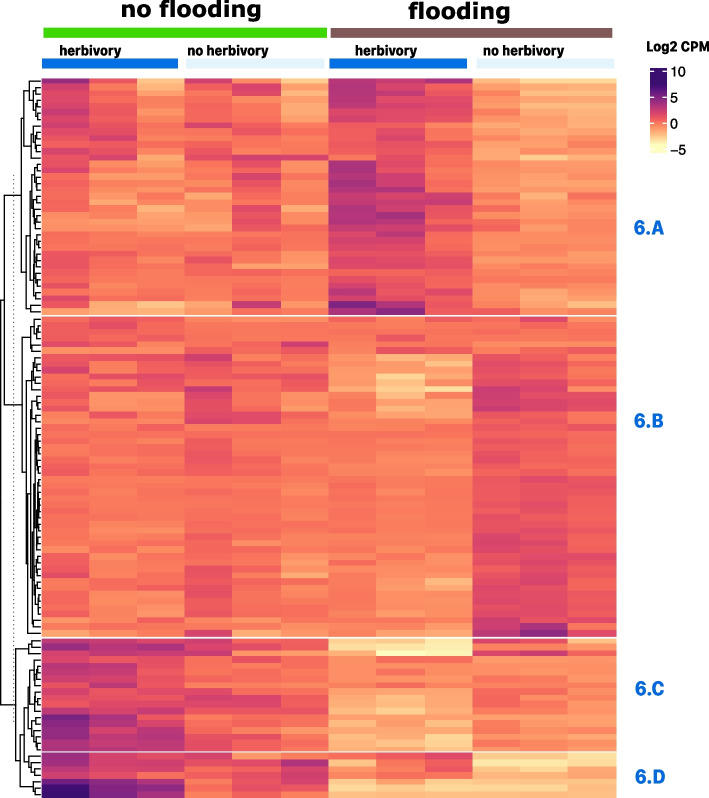
K-means clustering analysis of genes differentially expressed in leaf tissue of tomato plants challenged with herbivory and controls in the tomato variety “Cherokee Purple” (fold-change > 4 and; FDR *p*-value < 0.01). The heatmap has treatments as columns and genes as rows. The top right inset shows the color scale for log2 counts per million (CPM) values with purple tones for up-regulated genes and yellow tones for down-regulated genes. The vertical striped line on dendrogram shows the level at which the tree was cut to separate groups based on k-means. Each separate cluster was named according to figure number followed by and alphabetic character. Each cluster gene content is described in detail in the text

Within SG, there were 112 genes differentially expressed due to herbivory (fold-change > 4 and FDR corrected *p*-value 0.01). Clustering effectively separated herbivory and no herbivory samples but only within the flooding or no flooding groups (Fig. 6). Cluster 6.A shows genes that were upregulated with flooding and herbivory together but not with either stress alone. Seventeen out of 37 of the genes in cluster 6.A code for heat shock proteins (46%).

Cluster 6.B shows genes that were mildly downregulated by herbivory in flooded samples, including genes in primary metabolism, development, and photosynthesis (Table S3). Herbivory did not have apparent effects in non-flooded plants. Cluster 6.C contained 18 genes that were downregulated with herbivory in flooded samples but upregulated by herbivory alone. Among them were 2 genes encoding zeatin, *O*-glucosyltransferases, a GDSL esterase/lipase (see previous section), and several proteinases and proteinase inhibitors. Cluster 6.D, with 7 members, contains genes that were upregulated with herbivory, at a higher extent in non-flooded plants compared to flooded plants (Tables S1, S2, S3) Cluster 6.D included on gene encoding for a heat shock protein (hsc70), a thioredoxin (LOC101244843), and two peptidase/proteinase inhibitors (*mcpi* and LOC101246961).

## Discussion

Identifying plant varieties and cultivars that can tolerate co-occurring biotic and abiotic plant stressors is critical to maintaining crop productivity in the context of a changing climate. Understanding how plants prioritize and respond to combinatorial stresses at the physiological and molecular levels and identifying the key genes and traits that underly adaptive responses remains a fundamental task for crop improvement and breeding of climate-resilient crops. To date, our knowledge about how crop plants like tomato respond to the combinatorial stress of flooding and herbivory remains limited. Available studies show that plant responses to combinatorial stresses are complex and hard to predict. In this study, we investigated volatile production levels and gene expression in two varieties of tomato plants following exposure to flooding, herbivory, and their combination.

Volatiles produced by plants undergoing biotic and abiotic stressors have been widely documented. Individually, biotic or abiotic stress factors, such as herbivory [20, 44–46], elevated O_3_ [21], drought [20, 45, 47], and salinity [47, 48], are known to alter volatile emissions. Much less is known about how volatiles that mediate ecological interactions among plants, insects and their natural enemies are influenced by concurrent stress factors. Furthermore, results from the few studies that investigated the influence of combinatorial stresses on plant volatile emissions are variable. For instance, the combination of drought and feeding by the potato aphid increased volatile emissions in tomato [20]. Ngumbi and Ugarte [31] reported an increase in VOC emission in maize exposed to flooding and herbivory by the fall armyworm, *Spodoptera frugiperda*. By contrast, Tariq et al., [18] reported a decrease in the emission of volatiles when *Brassica oleracea* plants were subjected drought and root herbivory. In our study, we showed that individually, flooding induced greater total volatile emissions in one of the tomato varieties (SG), with no effect in CP. Herbivory alone significantly decreased VOC emissions in CP, with no significant effect in SG. Combined flooding and herbivory significantly increased VOC emissions in both varieties compared to the controls. These results are in agreement with studies that have reported increased emissions of volatiles in response to interacting biotic and abiotic stresses in plants of agricultural importance including tomato and maize [20, 30, 31]. There is evidence that the induction of volatile emissions in plants undergoing stress is part of a programmed plant response to alleviate and mitigate the negative consequences of stress [4, 49, 50]. In our study, we identified 18 volatiles, mainly terpenoids, including α-pinene, o-cymene, β-pinene, ( +)-4-carene, β-phellandrene, caryophyllene and humulene. Increased amounts of these compounds were detected in plants exposed to the combined stress of flooding and herbivory. These VOCs have been reported to play important ecological and physiological functions that ultimately increase plant fitness by attracting natural enemies of insect herbivores and relieving plants of ongoing oxidative stresses [51]. Plants that are exposed to heat, high temperatures, or drought, for example, have been documented to increase volatile emissions; released volatiles act as thermoprotectants and help in stabilizing chloroplast membranes [52, 53]. Alternatively, these induced volatiles serve as signal molecules that activate regulatory genes involved in stress tolerance and plant defense [54]. The ecological roles of the volatiles we identified to be increased in the combinatorial stress treatments remains to be determined.

In our study, most of the released volatiles, were mono- and sesquiterpenes. We did not collect green leaf volatiles. There are several possible reasons that could have contributed to the lack of detection of green leaf volatiles across the treatments. The quality and quality of the blend of volatiles that is released by stressed plants can be influenced by many factors including the method used to collect headspace volatiles, the time of sampling, the herbivore used to elicit volatile organic compound production, the genotype or variety of plant used, and the microbiome of the plant [44, 55–57]. It is possible that the tomato varieties we used in this study, Cherokee Purple and Striped German, may not be prolific emitters of green leaf volatiles. It is also possible that because we sampled volatiles 48 h after the herbivore was introduced, we missed the short window in which green leaf volatiles are emitted. Future studies will investigate time-dependent volatile emissions in tomato plants experiencing flooding, herbivory and the combinatorial stress of flooding and herbivory. Moreover, in our study, in the treatments that involved herbivory by *S. exigua*, we did not detect a strong response in volatile emissions and gene expression. The varieties used in this study may have high levels of constitutive defenses. In this study, we did not starve the insects before allowing them to feed on experimental tomato plants to induce herbivore-induced volatiles and gene expression. In future, we will starve insects for 6 h before placing them on plants.

Biomass measurements showed that flooding detrimentally impacted plant growth, in agreement with previous studies that have investigated the impact of flooding on plant growth characteristics [35, 58–60]. In general, below-ground tissues were affected by flooding but not by herbivory, whereas above-ground tissues were affected by flooding alone and flooding combined with herbivory. Reduced plant growth is a direct result of shutting down of primary plant processes such as photosynthesis, and alterations in soil nutrients availability [61, 62]. Ultimately, reduced plant growth leads to poor quality crops and a reduction of crop yields. Our results showing that flooding negatively impacts plant growth add to the growing body of evidence documenting the detrimental impacts of flooding and further emphasize on the need for more research to understand the negative impacts of flooding on agricultural crops including tomato.

Gene expression analyses indicate that some key differences between the two heirloom tomato varieties reside in secondary metabolism pathways. CP had higher constitutive levels of genes encoding Phenylalanine ammonia-lyase (PAL). PAL converts L-phenylalanine to trans-cinnamate and ammonia is the first committed step in the phenylpropanoid pathway, redirecting large amounts of fixed carbon from primary to secondary metabolism [63, 64]. Phenylpropanoid derived metabolites including anthocyanins, flavonoids, isoflavonoids, phytoalexins, lignans and terpenoids have important functions in plant resistance mechanisms against biotic and abiotic stress, in signal transduction and communication between plants and other organisms, and as regulators of primary and secondary metabolism [63, 65–70]. Differential PAL gene expression patterns in response to biotic and abiotic stressors can result in plant phenotypes that differ in their stress adaptive responses and stress tolerance levels [68, 71, 72]. Psy1, also showing higher expression in CP, catalyzes the first step in the carotenoid biosynthetic pathway. Our results showed that CP may have increased constitutive production of phenylpropanoid compounds and terpenoids. Huang et al., [68] demonstrated that wild type *Arabidopsis* plants and *pal* mutants that differed in their *pal* gene expression patterns exhibited remarkable differences in their adaptive responses and sensitivity to biotic and abiotic stressors.

Our goal was to characterize the interactions between different stressors. We identified a group of genes that responded to flooding in different ways in the two varieties. In general CP plants subjected to flooding showed a down-regulation of ABA production, SG, in turn appears to have constitutively higher expression of genes in either ABA biosynthesis or in downstream ABA signaling. Abscisic acid (ABA) is an important phytohormone that regulates plants growth and development [73] and has been reported to be a key regulator of plant responses to abiotic stresses [74] because its accumulation controls multiple gene response networks, ultimately leading to tolerance and adaptation to abiotic stress [28, 29, 73, 75–77]. Genes involved in ABA biosynthesis and catabolism could be activated or decreased by abiotic stress [78, 79]. Studies on the role of ABA in modulating plant responses to flooding report variable results. For instance, using wild type and ABA-deficient tomato plants, [29] demonstrated that flooding reduced ABA content in wild type tomatoes and suggested that depletion of ABA serves as a positive signal that leads to the induction of several specific genetic and metabolic responses to flooding. Similar results have been reported in other plants including in *Arabidopsis*, soybeans, and tomato [42, 80–84]. In contrast, an increase in ABA content and levels in response to flooding has been reported in leaves of alfalfa and pea [85, 86]. In our study, consistent with [29], we documented different expression patterns of genes associated with ABA signaling in the two tomato varieties. Results from our study further confirm the involvement of ABA in plant responses to flooding stress.

Flooding alone affected several hormonal pathways and photosynthesis. Consistent with our biomass data, the downregulation of photosynthesis genes in plants undergoing flooding stress has been documented, with a subsequent impact on plant growth [19, 29, 87]. Reduced photosynthesis in plants actively going through flooding stress can be correlated with the production of reactive oxygen species (ROS) and cell damage [88–90]. High concentrations of ABA and increased xylem pH have been correlated with reduced stomatal conductance [91]. High foliar concentrations of auxins [92] and ABA [93] may build up and create an accumulation message in leaf tissues of flooded plants due to stomatal closure, resulting from root oxygen deficiency initiating loss of root hydraulic conductance, or from an increase in pH [94]. Crosstalk between plant stress-response pathways likely reduces fitness costs, by reducing production of redundant hormones. Indeed, studies have shown gibberellic acid (GA) mediates gene expression of jasmonic acid (JA) [95], while JA and salicylic acid (SA) mediate immune response [96].

In our study, flooding initiated down-regulation of cytokinin-related genes, in addition to up-regulation of ethylene biosynthesis and multiple gene encoding enzymes used in the latest oxygenation steps of GA, which give rise to active gibberellins. Consistent with our results, flooding has been shown to depress cytokinin activity [97, 98], and lead to accumulation of ethylene [99, 100]. Ethylene is likely critical for plants to initiate signals that prolong their survival under abiotic stress [101]. For example, epinasty (downward leaf growth), a response to flooding in tomato, arises in response to signals from oxygen‐deficient roots and upregulation of 1‐aminocyclopropane‐1‐carboxylic acid (ACC), the immediate precursor of ethylene [102]. Ethylene-mediated ROS signaling is critical in regulating hormonal pathways that lead to adaptive formation of lysigenous aerenchyma, used for gas diffusion in roots during flooding [103]. Submergence-induced accumulation of ethylene has also been implicated in upregulation of internode elongation [100]. GA regulates plant growth and abiotic stress tolerance through mediating growth and stress responses to abiotic stress [104]. One mechanism, demonstrated in *Arabidopsis* under salinity stress by [105] showed growth-repressing DELLA proteins promoted survival, through downregulation of root-hair growth and reduction of reactive oxygen species (ROS) levels. In deep-water rice, Kuroha et al., [106] reported increased GA production upon plant waterlogging, underlying the adaptation of rice to periodic flooding. Plausibly, a similar signaling mechanism is utilized by heirloom tomato plants. Finally, flooding upregulated eight genes encoding heat-shock proteins Broadly, heat shock proteins are regarded as dynamic biomolecules that help plants to counter biotic and abiotic stresses [107] via several mechanisms including enhancing membrane stability and detoxifying the ROS by positively regulating the antioxidant enzymes system.

We studied herbivory by looking at the effects upon feeding of leaf tissue by *Spodoptera exigua*, a leaf chewing caterpillar. The effects of herbivory were mild, and only flooded plants had measurable response to herbivory in both varieties, with a very small set of genes upregulated by herbivory alone in only one of the varieties (SG). This indicates that flooding might weaken some aspects of the plant stress response while priming others. In CP, several stress response proteins were downregulated by herbivory (in flooded plants) but key genes in the phenylpropanoid pathway were upregulated. Among the few genes that were upregulated in CP flooded plants was also an UDP-glucose iridoid glucosyltransferase-like, suggesting the involvement of iridoid or iridoid-like terpenes in the response to herbivore in flooded CP plants. In SG, a disproportionately large number of heat-shock proteins were upregulated by flooding and herbivory together. Heat shock proteins are one of the important and significant molecular chaperones that play key roles in abiotic and biotic stress tolerance by actively participating in protein quality control, enhancing membrane stability, regulating diverse signaling pathways, and detoxifying the ROS [53, 107–114]. In agreement with previous studies, including studies done on maize, soybeans, and tomato, that have revealed the upregulation of various heat shock proteins by flooding, our results revealing an upregulation of heat shock protein genes, suggest that these proteins likely play a significant role in protecting proteins from denaturation and degradation and detoxifying ROS during flooding stress [115]. Studies on flooding in plants including maize, soybean and tomato have revealed that pathways involving various heat shock proteins are triggered and upregulated by flooding [90, 115]. Heat shock protein responses to biotic and abiotic stress and their combinations are shaped by the type of the insect herbivore, plant type and variety, and plants development stage when stress factors are applied [107].

Finally, as we were able to identify some specific VOCs and gene pathways that seem to be important in the plant response to flooding and the combination of flooding and herbivory, we should consider some of the factors and limitations that could have affected the results and prevent generalizations. For example, in our study the plants were first exposed to flooding before being exposed to herbivory, this order is not trivial since the plants can prime a defense response based on the first stress they encounter. In addition, is likely that the responses of the plants to a chewing insect are different from those to insects with different feeding strategies. Finally, the chemical profiling method for this study focused on volatile compounds; many, if not most, phenylpropanoid compounds that are part not only of the response to the stresses but as part of the constitutive differences between the two tomato varieties are not volatiles. With those considerations, this study sheds light into combinatorial stress responses and addresses our initial hypotheses; that the combined effects of flooding and herbivory differ from either stress alone, and that different tomato varieties can have better tolerance to those stressors.

## Conclusions

We demonstrated that flooding and the combination of flooding and herbivory elevated VOCs production, the majority of which were terpenes. Gene expression data demonstrated an upregulation of genes involved in the phenylpropanoid and terpenoid production pathways in at least one of the tomato heirloom varieties (CP) in response to these stresses. Plant volatiles mediate numerous plant and insect interactions [116]. How stress combinations alter plant VOCs can be better explained by looking at the genetic underpinning of the response to those stressors. Our study highlights the importance of studying combinatorial stresses and sheds light on key pathways that might be mediating this response as well as the complex dynamics that might at play during combined stresses. How these documented changes in gene expression patterns and plant chemistry affect plant and insect interactions deserves further investigation.

## Methods

### Tomato plant varieties

Two organic heirloom tomato (*Solanum lycopersicum* L.) varieties were used in this study; Cherokee Purple (CP) and Striped German (SG). We selected the heirloom varieties based on results from an unpublished survey by our lab of most popular tomato varieties grown organically by farmers in Central Illinois. Moreover, heirloom crops are prized by farmers, chefs and consumers for their diverse coloration, distinct flavor, and culinary qualities [117–120]. After performing numerous exploratory flooding experiments on four different tomato varieties commonly grown by Central Illinois farmers, we selected two heirlooms, Cherokee Purple (CP) and Striped German (SG) as most suitable to greenhouse experiments. CP grows relatively short vines, and produces a flattened, medium-large fruit, that is dusty pink with dark shoulders at approximately 72 days; the fruit has a rich, sweet flavor. SG grows medium-tall vines, and produces a flat, medium-large fruit, with variable shoulder ribbing in yellow and red at approximately 78 days; the fruit has a complex fruity flavor and smooth texture. Our selection was also informed by previous studies which indicate the broad genetic diversity found in heirloom tomato cultivars are better able to resist fungal pathogens [121], bacterial diseases [120, 122], and can maintain nutritional quality and yields under abiotic stressors such as drought [123] and salinity [124]. Seeds were obtained from Johnny’s seeds (Johnny’s Seeds, Winslow, Maine, USA). Seeds were germinated in seed trays (planting trays (model 1020), in 72-cell trays; 25 × 51 cm) containing potting soil (Berger BM2 Seed Germination & Propagation Mix; Berger, Saint-Modeste, Quebec, CA). Seedlings were grown at the greenhouse facilities of the University of Illinois at Urbana-Champaign plant care facility (PCF) at 25 °C ± 5 °C, 50 ± 5% relative humidity and 14L:10D photoperiod for two weeks.

### Insect herbivore

The beet armyworm, *Spodoptera exigua* (Lepidoptera: Noctuidae), a generalist herbivore caterpillar, was used as the herbivore species in this study. *S. exigua* caterpillars were purchased from Benzon Research (Carlisle, Pennsylvania, USA) and maintained in a constant temperature incubator at 26 °C and exposed to a 16L:8D photoperiod until the start of the experiments.

### Experimental design

Two weeks after germination, seedlings were transplanted into individual plastic pots (12.5 cm high, 16.5 cm diam) (Hummert International, Earth City, Missouri, USA) containing field-collected soil (Drummer silty clay loam) from Champaign County, Illinois, USA. One tomato seedling of each variety was transplanted into an individual pot. Plants were grown for three weeks at 25 ± 5 °C, 50 ± 5% relative humidity and 14:10-h (L/D). Three weeks after transplanting, plants were randomly assigned into four treatment groups: 1) no flooding + no herbivory, 2) flooding + no herbivory, 3) no flooding + herbivory, and 4) flooding + herbivory. Flooding was imposed by placing the pots containing tomato plants within a secondary larger white plastic bucket (16.5 cm high, 21.5 cm diam) (Consolidated Plastics, Stow, Ohio, USA) and filling it with water up to 5 cm above the soil surface. Plants in the no flooding treatments were watered regularly to maintain field capacity.

For the treatment factors involving insect herbivory, preliminary experiments showed that third instar *S. exigua* larvae acclimate and start feeding within 12 to 24 h of placement on a plant. Two third instar caterpillar larvae per plant were introduced three days post-flooding and allowed to feed for 48 h prior to volatile collection and collection of samples for gene expression analysis.

### RNA sequencing and analysis

Samples were taken from 40-day old tomato plants that were subjected to flooding for 5 days and/or herbivory for 2 days. Plants that were subjected to both treatments were exposed to herbivory on the last 2 days of the 5-day flooding period. Control samples consisted of 40-day old tomato plants with no flooding or herbivory. Samples were obtained by cutting the tip of the leaflet closest to the stem from the second true leaf, using sanitized micro-dissecting scissors (re-sanitized for each sampling). Samples were individually collected in sealable plastic sleeves, sleeves were heat sealed, and immediately flash-frozen by submerging in liquid nitrogen.

Total RNA from each sample was extracted using a Nucleospin® RNA kit (Macherey–Nagel, Düren, Germany) according to the manufacturer’s protocol. There were three biological replications per treatment (flooding or herbivory) across two tomato varieties. The RNA was sequenced as single reads on one S1 lane for 101 cycles on a NovaSeq 6000 (Illumina, San Diego, CA). After sequencing, reads were pre-processed, mapped to the tomato reference genome (SL3.0) using STAR aligner [125], and quantified for differential expression with the EdgeR Bioconductor package [126]. Clusters for all sets were generated with the K-means method in R (v. 4.1.1) using the Hartigan and Wong algorithm [127] with a maximum of 100 iterations and 10 initial random sets. The number of clusters (k) to partition the data was visually determined after generating heatmaps and dendrograms with the Complex Heatmap package from R Bioconductor [128]. Gene ontology analyses were performed using topGO with the ‘elim’ and ‘weight’ algorithms to prevent redundant GOs from inflating the significance [129].

### Collection and analysis of headspace volatiles

Aboveground headspace volatiles were collected using the solid phase micro-extraction (SPME) technique. Exhaustive preliminary experiments showed that the SPME method gave reliable and consistent results, that were comparable to other dynamic headspace volatile collection techniques. For the treatments without herbivore damage, plants were wrapped with an odor-blocking oven bag (Arcadia INTL, El Monte, California, USA) and wrapped for one hour to allow for volatile concentration. After one hour, a SPME fiber (65 µm polydimethdimethylsiloxane-divinylbenzezne (PDMS/DVB) fused silica, and stainless-steel fiber (Millipore Sigma®, Milwaukee, Wisconsin, USA) was inserted into each bag for 40 min, withdrawn, and then run immediately through coupled gas chromatography-mass spectrometry (GC–MS) for volatile identification and analysis. For the treatments involving herbivore damage, two third instar *S. exigua* larvae were allowed to feed on each tomato plant for 48 h before volatile collection. After 48 h with larvae still feeding, plants were wrapped with an odor-blocking oven bag, and similar headspace volatile collection protocols as described in treatments without herbivore damage were used.

Volatiles were identified using GC–MS, Hewlett-Packard (HP) 6890 GC (Hewlett-Packard, Sunnyvale, California, USA) in splitless mode, interfaced to an HP 5973 mass selective detector (MSD) with helium carrier gas. The GC oven was programmed as follows: inject at 40 °C, hold at 40 °C for 2 min, and then increase by 5 °C/min to 200 °C, for a total of 40 min. Injector and transfer line temperatures were 200 °C. Peak identification was performed using the NIST 98 library and by comparing published GC profiles of tomato headspace volatiles [130]. The structures of identified compounds were further confirmed by using synthetic standards that are commercially available purchased from Millipore Sigma® (St. Louis, Missouri, USA).

### Plant growth parameters

At the end of the experiment, shoots and roots were harvested. Fresh shoot and root weight was recorded, and the shoots and roots were placed in paper bags, oven dried at 70 °C for 3 days, and their dry weights recorded.

### Statistical analyses

Statistical analyses were performed in R software version 4.1.2 (R Core Team, 2021). Figures were produced using ggplot2 v.3.3.5 [131]. To visualize differences in total volatile organic compound emissions among treatments, non-metric dimensional scaling (NMDS) was used on a Bray–Curtis dissimilarity matrix using the metaMDS function of the vegan package v.2.5–7. The influence of variety, flooding, herbivory and their interactions on gene expression patterns, plant volatile organic compound emissions and plant growth characteristics were analyzed and compared using a three-way analysis of variance (ANOVA) followed by Tukey–Kramer HSD at *P* < 0.05. To identify the set of important volatile compounds that distinguish among treatment combinations, Random Forest (RF) algorithm for classification as outlined in [132, 133] was used. The RF analysis was performed using the package “randomForest” within the R environment using the function randomForest [134]. The model ran for 1,000 iterations for variable selection. VOCs importance was ranked with mean decrease in accuracy (MDA), where a higher MDA indicates higher importance in classification. The RF analysis generates the average out-of-bag (OOB) error, with a low OOB error suggesting a greater ability of the variable to differentiate treatment factors.

## Supplementary Information

**Additional file 1.**

**Additional file 2.**

**Additional file 3.**

**Additional file 4.**

## Data Availability

All raw RNA sequence data are available a NCBI under project accession: PRJNA850397.
